# Product News

**DOI:** 10.1155/S1463924679000493

**Published:** 1979


### Product News

### Titrator

Baird & Tatlock have announced an addition to their range of Karl Fischer titration equipment. The titrator, the AF3, is a fully-automated, push-button system. It comprises two basic modules. A metering module houses the controlled impulse metering pump and liquid handling system. It automatically takes the correct volume of reagent straight from a standard Winchester bottle. A titration module contains the electronics and main controls and automatically displays the results directly as a digital read out in milligrams of water to the nearest 0.11 mg. All results are compatible with earlier Karl Fischer titrometric units.

*Baird & Tatlock (London) Ltd., PO Box 1, Romford, RMl 1HA, UK.*

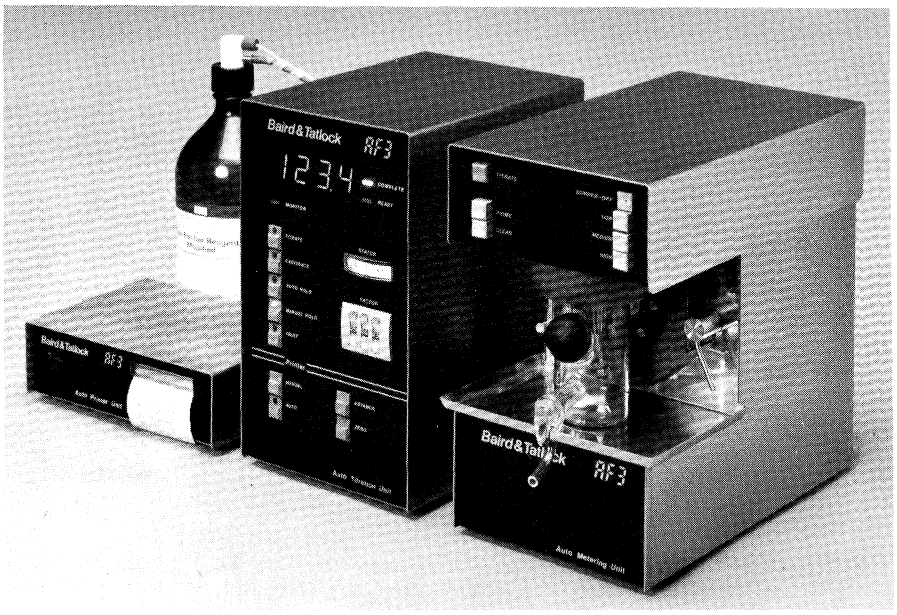

*The AF3 direct reading Karl Fischer titrator*

### Carbon dioxide analyser

Corning Medical have introduced a total carbon dioxide analyser which operates on an automated adaptation of the Van Slyke method enabling routine or emergency $TCO_2$ determinations to be carried out rapidly. The analyser accepts samples of 50-100 microlitres and uses one millilitre of reagent per test. Readout is linear within ± mmol/l over the full 0-60 mmol/l $TCO_2$ range. Reproducibility is claimed to be 1.7% C.V. at 30 mmol/l, as determined by measuring 10 replicates of pooled sera or aqueous standard solution. The instrument weighs 9 kg and is 330 x 388 x 234 mm in size. It operates on standard electrical supplies of 90-125 or 190-250V, 50 or 60 Hz.

*Corning Medical-France, 11 chemin de Ronde, 78110 Le Vesinet, France.*

### Enzyme immunoassay

The Abbott CEA-EIA system is a solid phase enzyme immunoassay. Beads coated with anti CEA are incubated with the specimens (standards, controls and unknown). CEA present in the specimens is then bound to the solid phase. Unbound material is removed by washing the beads. Subsequently anti CEA conjugated with horseradish peroxidase is incubated with the beads and if CEA was present in the specimen, the enzyme-labelled anti CEA is bound to the beads. Unbound conjugate is removed by washing. On incubation with the enzyme substrate a yellow colour develops which measures the amount of bound anti CEA-peroxidase conjugate. After stopping the enzyme reaction by addition of 1 M hydrochloric acid, the intensity of the colour developed is read using a spectrophotometer set at 492 nm. Within the working range of the assay the absorbance is proportional to the concentration of CEA in the specimen.

*ABBOT GmbH, Diagnostics Division, Amperestrasse 3-5, 6070 Langen, 06103/701-1, West Germany.*

### Waveform processor

Datalab have introduced the DL417, a microprocessor-based calculator for use with its DL Micro 4 Signal Analysis System. The calculator avoids costly complexity of the minicomputer method but allows the module to be used for a wide range of signal averaging and waveform analysis problems beyond the capability of the average keypad size.

The existing system is a modular instrument with a data highway connecting one of a range of signal analysis modules to a timer unit, a large digital memory and a display controller. The microprocessor in DL 417 is furnished with a number of resident programs which are either functions or trigonometric operations or routines. By means of a highlevel user oriented language, two or more standard functions can be combined into an expression which will perform an operation on data from a selected channel. The analogue waveforms and alphanumeric characters are displayed on an external CRT. Waveforms can be selected for intensification, expansion and manipulation. The display can be divided into two or four separate channels, each of which can be sub-divided further for processing purposes.

*Data Laboratories Ltd, 28 Wates Way, Mitcham, Surrey, CR4 4HR, UK.*

### RIA test system

Bio-Rad Laboratories have introduced the Quantimune Digoxin RIA test system based on the solid state support technique using immuno-beads as the support. Quantimune Digoxin is immediately available in reagent packs of 100 and 200.

A comprehensive range of assays in the field of lipid chemistry are also available from Bio-Rad laboratories. These include the HDL/Cholesterol assay, Quantazyme Cholesterol, Quantazyme Triglycerides and the Bio-Gram A Lipoprotein electrophoresis test system. The HDL Cholesterol system comprises a one vial, predispensed, lyophilised separation system. Both the cholesterol system and triglycerides assay systems are single vial, fully enzymatic, easily automated end-point assays. The disadvantages of multivial reconstitutions, additions and critical timing are thereby eliminated.

*Bio-Rad Laboratories Ltd, Caxton Way, Holywell Industrial Estate, Watford, Herts WD1 8RP.*

### Data acquisition cassette system

A microprocessor-controlled cassette unit, the MFE 25000 is now available from Data Dynamics. It has been specifically designed for use in data acquisition and off-line data capture. Data is recorded onto cassette in standard ANSI/ECMA format with each record comprising: preamble, 86 data characters, CRC check character, and post-amble.

The unit has two serial ports which would normally be connected to a keyboard terminal and a CPU or modem. The terminal port can be RS232C or 20 mA current loop. The modem port is RS232C. Communication rate is at 110, 300, 1200 or 2400 baud, as pre-set on a selector switch. It can be manually controlled by front panel switches or remotely controlled by ASCII control characters received from line. Front panel controls are: send, receive, local copy, binary, on-line, rewind and baud rate. Various other remote controls are incorporated.

Each cassette will store 2,000 86-character formatted records per side, which are recorded at 800 bits per inch. Read and write operations are accomplished at a speed of 12 inches per second, and rewind at 80 inches per second.

*Data Dynamics, Data House, Springfield Road, Hayes, Middlesex, UK.*

### Titration system

Metrohm of Switzerland have developed an automatic microprocessor-based titration system which has been introduced to the British market by Roth Scientific Ltd. The system, the E636 titroprocessor offers fully automatic operation in chemical titrations with full calculator facilities.

Operation is simple and requires no previous knowledge of programming techniques. The instrument controls the rate of titrant addition in accordance with the corresponding changes in the value measure by the electrode chain. Sensitivity, speed, reaction kinetics and other normal parameters involved in titration procedures can be programmed into the unit. This operation is effected by means of the key-board or by special cards that can be marked by the operator. If no information is fed in, then the microprocessor uses built-in standard values.

The instrument cannot go 'off scale' because even if the wrong measuring parameters are chosen the normal curve can be recalled as often as required, with any necessary amendments to the selected measuring scales included.

Full titrations or titrations to a pre-set end point can be performed. The unit can be stopped after a pre-selected number of end points are found, or at a given mV, pH or ml value. Results are printed out in ml, with the mV or pH value at each end point and the mV or pH value at the start of the titration. A calculation card can be read in to the instrument in order that the millilitre results found can be converted into any units desired.

The burette is fully automatic and accepts complete plug-in burette assemblies of 1, 5, 10, 20 or 50 ml capacity. All parts in contact with the titrant are glass or Teflon and the burette tap is of a micro column, flat disc, self lubricating Teflon design. The integral reservoir is of 1 litre capacity.

*Roth Scientific Co. Ltd., Zurcourt House, Osborne Road, Farnborough, Hants.*

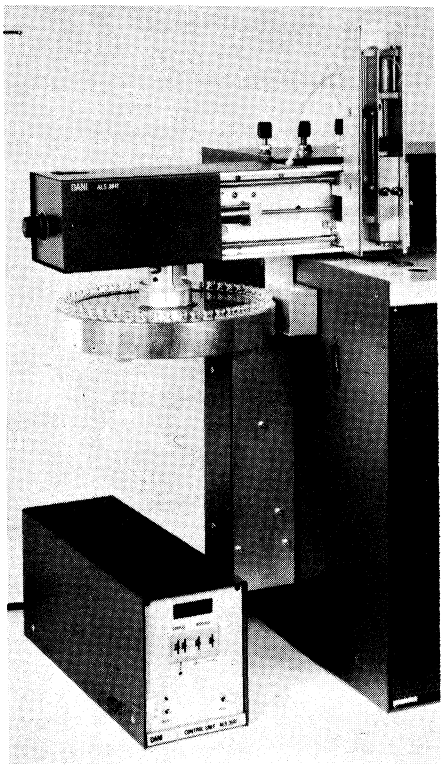

*The Dani ALS 3641 automatic sampling unit for GC*

### Automatic sampling unit

An automatic sampling unit, the ALS 3641, has been developed by Dani which automates the metering and the injection of liquids into every gas chromatograph provided with vertical injection port. The carousel can house up to 50 sample vials which are sequentially presented at the sampling probe. A standard microsyringe is used to meter the sample volume. The automatic mechanism duplicates the essential operations and step-by-step technique of the experienced analyst. It flushes the syringe several times with the sample, aspirates a precisely measured sample volume and injects a reproducible slug into the gas chromatograph. In order to prevent evaporation of the sample in the needle, an air plug is aspirated to reproduce exactly the operation of a skilled chromatographer. After the injection is completed, the syringe is flushed again with solvent in order to remove any trace of the previous sample and it remains clean waiting for a new sample. The number of washing cycles with solvent and/or with sample are selected by the operator on thumbelwheels; the number of the vials in analysis is displayed, and the vial number can be requested by computer for repetitive analysis. Other products introduced by Dani include the 6800 gas chromatograph and various accessories for gas chromatographs.

*Dani SpA, Via Rovani 10, 20053 Monza (MI), Italy.*

### Block digestor system

Techne have introduced two new products to their range of interchangeable block heaters. The DG-1 Block digestor system is designed for Kjeldahl, COD and other wet digestion or oxidation techniques. This unit is a complete package of block heater with modular, digital set, temperature controller, combined tube rack and heat shield, a set of tubes, and bench stand. The block is interchangeable to take either 20 x 42 mm diameter tubes (250 ml capacity) or 40 x 26 mm diameter tubes (75 ml capacity). The temperature range is from 100°C to 450°C with a control stability at 400°C of ± 1°C.

The SC-3 sample concentrator system gives the user maximum flexibility in the preparation of samples prior to analysis. Gas is blown via a gas reservoir through hypodermic needles into the neck of the sample tubes. The tubes are heated by the block heater which operates between 30°C and 100°C with a control stability of ± 0.25°C. The blocks themselves are fully interchangeable to accept standard glassware and tubes. The gas reservoir may be raised or lowered to suit the level of liquid in the sample tubes so that the system can be operated at maximum efficiency whatever the sample size. Applications include drug screening, hormone assays, organic solvent analyses and other techniques requiring sample concentration.

*Techne Cambridge Ltd, Duxford, Cambridge, CB2 4PZ, UK.*

### Microcomputer design kit

GEC Semiconductors have introduced the Intel system design kit using the 8086 16-bit microprocessor. The kit, the SDK-86, is designed for engineers to gain first-hand experience of the hardware, architecture, and the machine code of the 8086.

It is a complete MCS-86 microcomputer system on a single board in an easy-to-handle kit form; and it contains all the necessary components to build a functional system quickly. A complementary system monitor is also supplied with general sottware utilities and system diagnostics in pre-programmed ROMs.

The SDK-86 communicates through either the on-board LED display/ keyboard combination, the user's TTY

or CRT terminal, or a special mode in which an Intellec development system can transport finished programs to and from the SDK-86. The memory capacity can be easily increased by soldering-in additional devices in locations provided for this purpose.

A manual is provided with the kit containing step-by-step instructions.

*GEC Semiconductors Ltd, East Lane, Wembley, Middlesex HA9 7PP, UK.*

### Sample injection valves

Altex has introduced a new idea in sample injection devices for high performance liquid chromatography. It is based upon a four-port valve design rather than the conventional six-port. The device is constructed in 316 SS and chemically inert polymers and has been designed for operation at pressures up to 10,000 psi, without generating wear particles which plug columns and filters.

The design provides easy access to all fittings and requires little force to actuate from the load to inject positions. Sample loops from 5 to 2000 μl are available, and smaller samples can be injected by partially filling the loop with a standard chromatographic syringe.

*Altex Scientific, Inc, 1780 Fourth Street, Berkeley, California 94710, USA.*

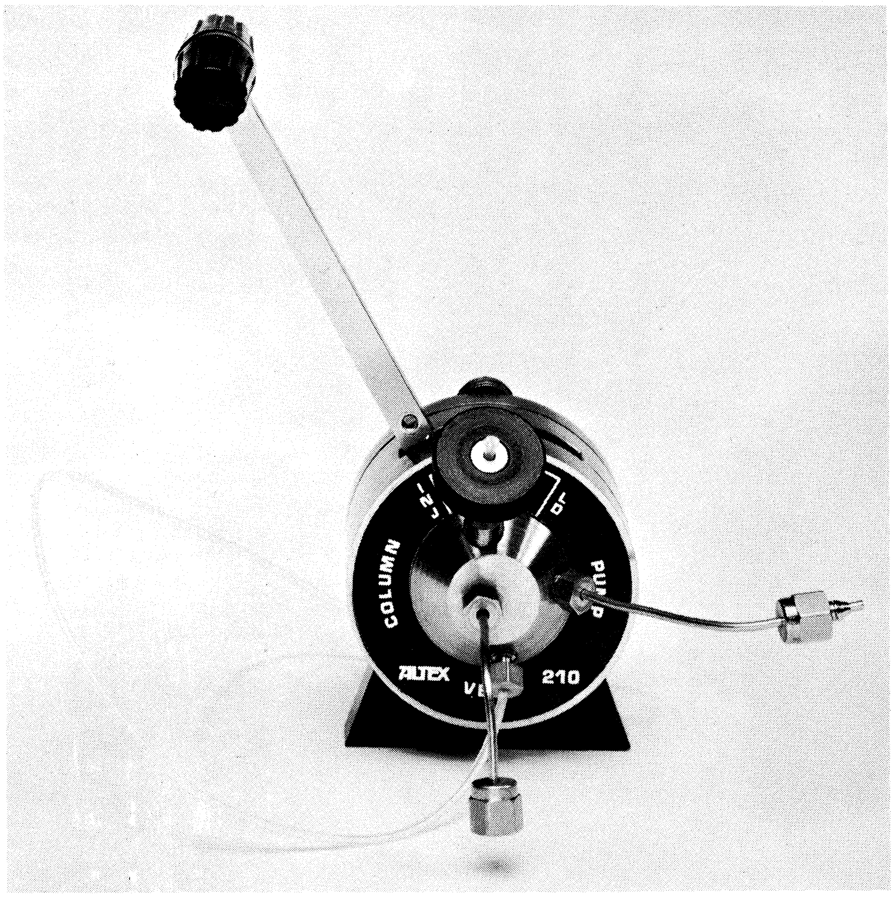

*The Altex Series 210 four-port sample injection valve for HPLC*

### Data acquisition systems

Perex Ltd have introduced the Perifile 8041 and the Perifile 8051 as the beginning of a new Series 8000 product range of microprocessor-based data acquisition systems. Both systems require 12 volts DC supply and are ANSI and ECMA 46 compatible. The DC300A magnetic tape cartridge is used for data storage. They comprise four printed circuit cards located in the rear of the drive chassis which conform to the Euro card dimensional standard. Each unit has a dual byte buffer that ensures that the data transfer to and from the data cartridge is transparent to the user. Data is logged back sequentially over the four tracks and if required a single or double tape mark may be written to tapes.

Data integrity is ensured by generation of a cyclic redundancy check work. If a read after write error is detected the block is erased and rewritten six inches further along the tape. If after four attempts an error condition still exists a read CRC is flagged. A write CRC override facility is available for the user.

The Perifile 8041 is a file orientated storage device which is capable of storing data in sequential files, each file identified by a file number. Both units feature either the EIA RS232 interface with data rates from 110 baud to 9600 baud or 8 bit TTL compatible parallel interface.

*Perex Ltd, Arkwright Road, Reading, Berks RG2 0LS, UK.*

### Computing integrator

Spectra-Physics has announced the introduction of their SP 4100 Computing Integrator. Features include automatic integration, built-in BASIC programming, an X-Y plotting capability, intelligent dialogue, and a range of pre-programmed data reduction methods. It has been designed primarily for use with gas or liquid chromatography systems. It is microprocessor controlled and features an alphanumeric keyboard, a full width LED display and a printer-plotter output. The BASIC programming capability allows the user to modify existing data reduction methods, to develop special reports or applications, and even to reprogram the standard instrument ROM software.

Many data reduction methods used in chromatography are pre-programmed in permanent memory (ROM), including area per cent, area normalisation, external standard, and three variations of internal standard. In addition to the keyboard, the instrument has 15 function keys labelled in familiar chromatographic terms which can all be redesignated by the user to suit his specific requirements. The LED display above the keyboard indicates verified entries and integration status.

The unit also offers such capabilities as measurement of peak heights or areas, editing of files while operating, true raw data chromatogram recording and playback using any hi-fi cassette deck, and optional timebase control by external events such as drop counter. It can be interfaced to the SP 4000 multi-channel data system, substantially extending the capabilities of both systems.

*Spectra-Physics Ltd, 17 Brick Knoll Park, St. Albans, Herts. UK.*

### Nitrogen analysis system

Antek Instruments, Inc has announced the introduction of its Model 703B Nitrogen System, a system designed for the determination of bound nitrogen in virtually any medium.

The system utilizes the chemiluminescent detection method to sense the presence of nitric oxide. The samples are oxidatively pyrolized in a furnace and then flow into the 703B. The chemiluminescence resulting from a flameless reaction between nitrous oxide and ozone is then monitored through a filter by a photo multiplier. A digital counter is located on the front panel. Additional features of this model include a diagnostic display with lights indicating operational discrepancies, digital display of signal integration, and analog output for 0-1 V recorder.

The system has been designed to operate with the Model 771 Pyroreactor, but it can be easily adapted to operate with other oxidative pyrolysis.

*Antek Instruments, 6005 North Freeway, Houston, Texas 77058, USA.*

### Liquid chromatograph

A high-performance liquid chromatograph (HPLC), the Series 750, suitable for demanding, high pressure analytical separations and medium flow semi-preparative separations, has been introduced by the Schoeffel Instrument Division of Kratos, Inc. The system is modular and can therefore be added to expand the capabilities.

It features a dual piston, high pressure pump, a high pressure gradient programmer, a column oven, and a multi-wavelength UV detector. The components may be used independently or together as a complete system. Constant volume mobile phase delivery is accurately provided from 0.1 to 9.9 ml/min, with less than 1% pulsations peak-to-peak. Two pumps are used for gradient elution, with full programmability over gradient formation. Pressure limit with the system is 5500 psi. The UV detector employs a deuterium lamp and provides high sensitivity at 200, 215, 240, 254 and 280 nm.

Options include variable wavelength absorption and fluorescence detectors, injection valves, recorders, integrators, all of which are compatible with this series.

*Schoeffel Instruments Division, Kratos Inc, 24 Booker Street, Westwood, NJ 07675, USA.*

### Oxygen electrode

The latest electrode in the Orion range of chemical sensing electrodes is the model 9708 dissolved oxygen electrode, which is now available from MSE Scientific Instruments. This electrode simplifies the measurement of dissolved oxygen, especially biological oxygen demand. The current produced in proportion to the oxygen concentration is converted to potential by means of a small electronics package built into the electrode. As the biological oxygen demand measurements fall within the range 0 to 14 ppm the pH scale of a pH meter can conveniently be used; the electrode can therefore be used with any pH meter.

*MSE Scientific Instruments, Manor Royal, Crawley, West Sussex, RH10 2QQ, UK.*

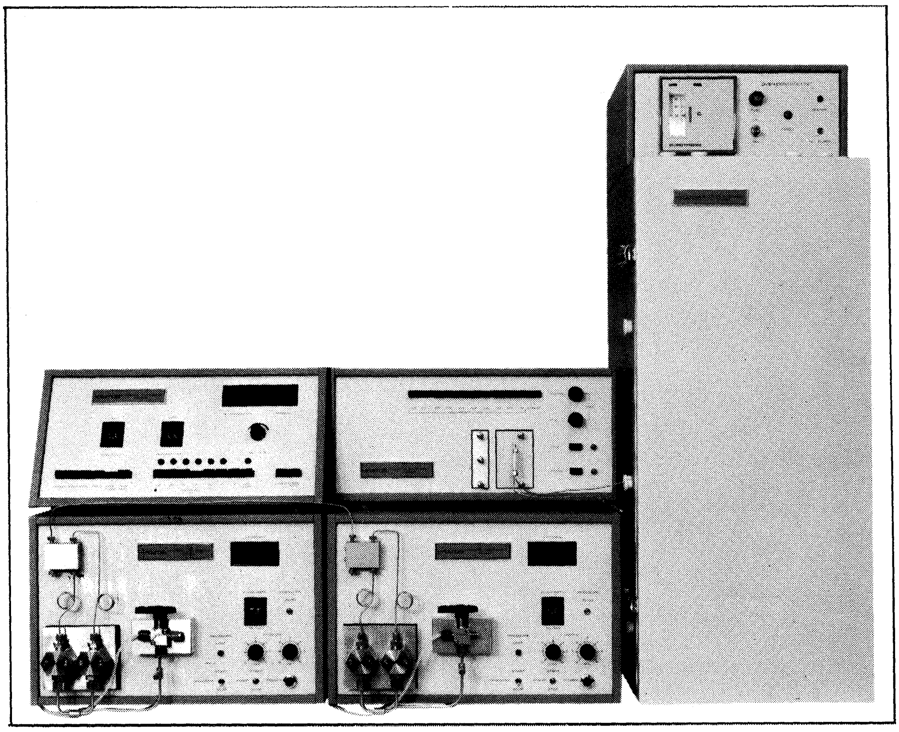

*The Series 750 HPLC system*

### Spectrophotometers and chromatographs

Pye Unicam have recently launched a new range of spectrophotometers and chromatographs. The atomic absorption flame system SP9 with computer control permits fully automated analysis of routine samples. The SP9 computer provides data handling capacity to meet many application needs. Facilities include curve correction, employing up to five standards in fixed or variable ratios, peak height or peak area, running mean mode and automatic warnings of over calibration or excess curvature. The flameless atomic absorption system SP9 incorporates the new microprocessor controlled furnace programmer, video display unit and furnace autosampler. The furnace programmer provides complete microprocessor control of the furnace and is equipped with autosampler controls. It can store up to ten complete atomisation programmes with either linear or non-linear temperature ramp, gas stop and temperature or voltage control.

The SP3 series of infrared spectrophotometers offer the performance benefits of a ratio recording system normally associated with high cost research type instrumentation. The SP3-100 is the most simple version in the series and has a wavenumber range of 4000 $cm^{-1}$ to 600 $cm^{-1}$. It is designed to meet applications where rapid sample throughput and quantitative accuracy are essential. The SP3-200 and SP3-300 models offer additional performance and flexibility. The latter covers the range 4000 $cm^{-1}$ to 200 $cm^{-1}$ with two diffraction gratings. These instruments are equipped with the company's 'spectraset' system.

Master holographic gratings have been incorporated in all their UV/visible spectrophotometers which significantly reduces the stray light. The SP6 series has been redesigned and the wavelength range has been extended to 190-1000 nm. The series includes the SP6-350 visible range and the SP6-550 UV/visible range models, each with a digital readout and an automatic four cell changer, an auto-cell, a SP4-01 automatic sample changer and HP975 software system for EMIT analysis. The SP8-150 has a stray light specification of 0.01% at 220 nm and the SP8-250 a specification of 0,0002% over the whole wavelength range.

Another new instrument range is the LC-XP Series of high performance liquid chromatographs. The series has a modular design approach which enables fully integrated systems to be supplied to suit specific requirements. The simplest configuration is the LC-XP single piston pump with the LC-UV detector together with column and injection systems. For more advanced performance there is a series of isocratic and gradient elution systems based on the LC-XPD dual reciprocating pump. Gradient elution programmes are simply entered by keyboard; stored and later retrieved and edited, as desired.

*Pye Unicam Ltd, York Street, Cambridge CB1 2PX, UK.*

### Data logging device

Computing Techniques Internations have introduced a single channel, multipurpose data logging device, the Lympet Logger.

It is fully portable, contained in a heavy duty, moisture proof case with a lockable hinged lid and is powered by rechargeable batteries. The life to discharge is a minimum of 60 days. There are two recording modes. Mode one will record 256 or 512 readings and then stop. Mode two will continue recording and over-writing previous data so that when readings are extracted only the previous 512 recordings are available.

Measurement can be taken from a wide variety of transducers. The logger is supplied with a fail safety switching so that once recording has started, data can only be lost by removing the battery. A translator is used for the examination of Lympet data and for calibration purposes. It gives a visual display of data stored and a teleprinter can then be used to provide a permanent printed record.

A manual is available which gives comprehensive information concerning the specification and potential applications of the product.

*Computing Techniques (International) Ltd, Brookers Road, Billingshurst, Sussex RH14 9RZ, UK.*

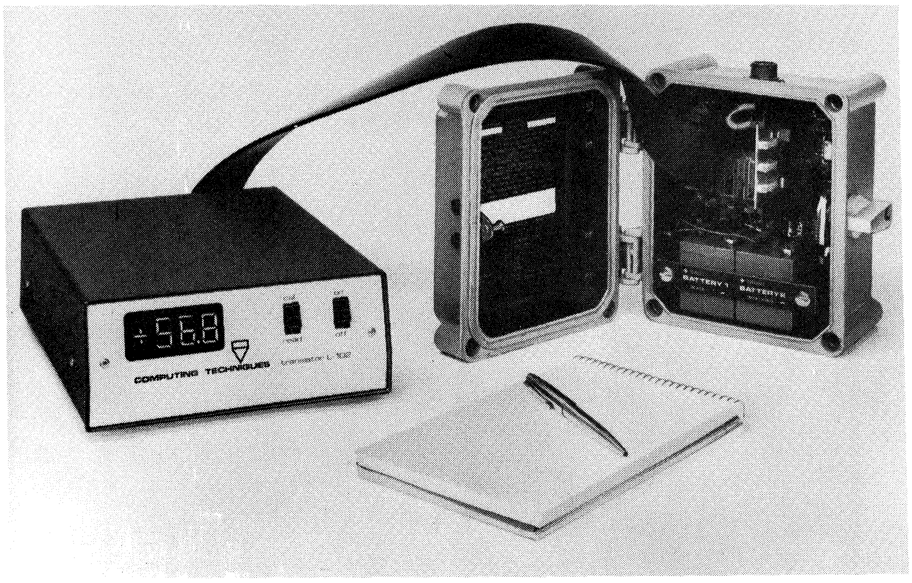

*The Lympet data logging unit connected to the translator*

### Laboratory computer systems

A new member of the 'MINC' family of budgetary laboratory computer systems has been announced by Digital Equipment Company's Laboratory Data Products Group. The system, designated DEClab-11/MNC, has both greater on-line storage capacity and increased program flexibility than the entry-level MINC system. It employs twin RLO1disk storage units for a standard 10-megabyte capacity and its fundamental programming is done in ANSI-standard FORTRAN IV, supplemented with laboratory subroutine packages. It is housed in a 104 cm high cabinet.

The design permits the user to customize the systems configuration through use of plug-in 'MINC modules'. The system will accept up to eight modules, which can include various combinations of the seven different functional module types. The modules include both interfacing and control types. The central element of the system is the 'MINC box' into which the modules plug and the central sub-assembly incorporates a PDP-11/03 microcomputer with 64K bytes of semiconductor memory. Other system elements include the twin removable storage disks, a 4-port terminal interface, a read-only memory (ROM) diagnostic bootstrap, and either an LA36 DECwriter II or a VT105 video terminal, which is capable of displaying both alphanumeric and point-plot or histogram waveform data.

An optional addition for the system is an IEEE-488 bus interface, which supports up to 14 laboratory instruments and testing devices conforming to the IEEE standard.

*Digital Equipment Co Ltd, Digital House, Kings Road, Reading, Berks, UK.*

### Solvent delivery system

A solvent delivery system for HPLC has been developed by Micromeritics Instrument Corporation. The 750 Solvent delivery system is a dual-piston, reciprocating design. It incorporates a patented cam and electronic flow multiplexing synchronization to achieve a high degree of flow precision. Typical flow precision is claimed to be better than ± 0.5% at flows from 0-20 ml/min and pressures to 6,000 psi. The absence of pulse dampeners also provides a reduction in total system volume which allows for single-pump, low pressure gradient programs to be formed with minimum lag time. Low volume makes unattended recycle more efficient. All major pumping system components are easily accessible through the front panel to facilitate maintenance operations. The system is universally adaptable to other HPLC components and may be upgraded to microprocessor control.

*Micromeritics Instrument Corporation, 5680 Goshen Springs Road, Norcross, Georgia 30093, USA.*

### Nitrogen analysis system

The model 707, a new digital nitrogen system designed to provide accurate detection of nitrogen in virtually any medium in as little as 30 seconds, has been introduced by Antek Instruments, Inc.

Typical applications for the Model 707 include analysis of bound nitrogen in wastewater, sewage, petroleum, petrochemicals, polymers, foods and beverages, fertilizers, drugs, pesticides, biological specimens, soils, coal and many others.

The new nitrogen system utilizes the chemiluminescent detection principle to sense the presence of nitric oxide, thus eliminating the need for the expense reduction method or the time consuming Kjeldahl method of nitrogen analysis.

The sample is introduced by means of a ceramic boat, pyrolzate (nitric oxide) and ozone is monitored by a light sensitive detector, providing highly specific measurement of total chemically bound nitrogen. Pyrolysis temperature is accurately controlled by a microprocessor assuring maximum conversion and repeatibility.

The model 707 can accommodate samples up to one gram in size and has a sensitivity of 1 nanogram with a dynamic range of $10^6$ With the extended range accessory (Model 732) the system can provide measurement of combined nitrogen in the percentage range.

*Antek Instruments, Inc., 6005 North Freeway, Houston, Texas 77076, USA.*

### Automatic clinical analyser

A third-generation automatic clinical analyser, the 'aca' III, featuring advanced microprocessor technology, will be shown for the first time in the UK by the Du Pont Company at the Third European Clinical Chemistry Congress in Brighton.

Currently under evaluation in the new 'aca' III incorporates the same charactistics as the present 'aca' instruments.

Additional features include, computer-assisted method calibration, automatic instrument standardization, instrument diagnostic programs, expanded report format, and alphanumeric keyboard and display.

Operator efficiency is greatly increased. The 'aca' III has the capacity to allow new methods to be added as they are developed by Du Pont. Vital parameters are displayed in a selected language to aid instrument operation. The instrument is compatible with any on-line laboratory information system.
*Du Pont de Nemours SA, rue de la Fusee 100, Mercure Centre, 1130 Brussels, Belgium.*

### New methodologies

A number of new diagnostic methods and program cards are now available on the Vitatron PA800 programmable analyser. Software has now been written to enable the user to assay immunoglobulins by the Boehringer Tinaquant methods, coagulation factors, as produced by Kabi and Boehringer and an extended number of EMIT procedures, as produced by Syva. A water filled reagent dispensing system, which requires no priming, ensures minimal expenditure on diagnostics and the programmable Canon calculator process data and produces hard copy data in real concentration terms, from typically non-linear calibration curves.
*MSE Scientific Instruments, Manor Royal, Crawley, West Sussex, RH10 2QQ, UK.*

*Editor's Note:-*
*The address of the manufacturer appears in italic at the end of each item. In some cases this address will be that of a subsidiary to the manufacturing company as the address given is that from which the information has been obtained.*

### Calendar

**Editor's Note:**
*Organisers of conferences, seminars etc. should send details for inclusion in this calandar as soon as the relevant information is available and not later than three months before the event.*

### 1979

**3rd International Symposium on Capillary Chromatography**
April 30-May 3, Hindelang/Allgau, W. Germany.
*R.E. Kaiser, P.O. Box 1308, D-6702 Bad Durkheim 1, Germany.*

**Ninth Annual Symposium on the Analytical Chemistry of Pollutants**
May 7-9, Jekyll Island, U.S.A.
*Mrs. Elaine McGarity, U.S. Environmental Protection Agency, Environmental Research Laboratory, College Station Road, Athens, Georgia 30605, U.S.A.*

**Current Developments in the Clinical Applications of HPLC, GC and MS**
May 30-June 1, Harrow, U.K.
*Symposium Secretariat, Division of Clinical Chemistry, Clinical Research Centre, Harrow, Middlesex, U.K.*

**3rd European Congress of Clinical Chemistry.**
June 3-8, Brighton, U.K.
*Dr. P.J.N. Howarth, Department of Chemical Pathology, Guy's Hospital, Medical School, London SE1 9RT, U.K.*

**2nd World Chromatography Conference**
June 5-6, Lisbon, Portugal.
*V.M. Bhatnagar, P.O. Box 1779, Cornwall, Ontario K6H 5V7, Canada.*

**10th International Symposium on Chromatography and Electrophoresis**
June 19 - 20, Venice, Italy.
*Dr. Alberto Frigerio, Instituto di Riccerche Farmacologiche "Mario Negri," Via Eritrea 62, 20157 Milan.*

**8th International Conference on Atomic Spectroscopy**
July 1 - 6, Cambridge, U.K.
*Association of British Spectroscopists, P.O. Box 109, Cambridge CB1 2HY, U.K.*

**2nd Canadian World Chromatography Conference**
July 5 - 6, Toronto, Canada.
*V.M. Bhatnagar, P.O. Box 1779, Cornwall, Ontario K6H 5V7, Canada.*

**Summer School on Automatic Methods of Analysis**
July 9 - 13, Swansea, U.K.
*D. Porter, Laboratory of the Government Chemist, Cornwall House, Stamford Street, London SE1 9NQ, U.K.*

**Analysis '79: Automation in Industrial and Clinical Chemistry.**
July 16 - 18, London, U.K.
*Scientific Symposia Ltd., 33-35 Bowling Green Lane, London EC1R 0DA, U.K.*

**Automatic methods in connection with Trace-organic Analysis**
September 4 - 7, Guildford, U.K.
*Dr. E. Reid, Wolfson Bioanalytical Centre, University of Surrey, Guildford GU2 5XH, U.K.*

**Automation and Computerisation in the Medical Laboratory**

September 5 - 7, Stirling University, Scotland.

*G.W. Thomson, Secretary, IMLS Glasgow Branch, 67 Glen Ogilvie, St. Leonards, East Kilbride, Scotland.*

**International Conference on Flow Analysis**
September 11 - 13, Amsterdam, The Netherlands.
*Secretary FA-Amsterdam, Laboratory for Analytical Chemistry, University of Amsterdam, Nieuwe Achtergracht 166, 1018 WV Amsterdam, The Netherlands.*

**Communications in Microprocessor Industrial Instrumentation**
September 12 - 13, London U.K.
*Mrs. P. Keiller, Sira Institute, South Hill, Chislehurst, Kent BR7 5EH, U.K.*

**Ninth International Meeting on Organic Geochemistry**
September 17 - 20, Newcastle-upon-Tyne, U.K.
*Dr. A.G. Douglas, Organic Geochemistry Unit, Geology Department, Drummond Building, The University, Newcastle-upon-Tyne, NE1 7RU, U.K.*

**Le Chromatographe Automatique Industriel en Lague: Analyseur Sophistique ou Capteur Industriel?**
October 3 - 5, Arles, France.
*Institute de Regulation et Automation Guy Berthier, Chemin des Moines, 13644, Arles, France.*

**Real-time Datahandling and Process Control**
October 23 - 25, Berlin, West Germany
*Congress Organisation Company, John Foster Dulles Allee 10,D-1000 Berlin,West Germany.*

### 1980

**Automation at SAC 80**
July 20 - 26, Lancaster, U.K.
*The Secretary, Analytical Division, The Chemical Society, Burlington House, London, W1Y 0BN, U.K.*

### 1981

**Euroanalysis IV**

August 23 - 28, Helsinki, Finland.

*Association of Finnish Chemical Societies. Executive Secretary, Pohj, Hesperiankatu 3B10, SF-00260 Helsinki 26, Finland.*

